# Descemet membrane detachment in femtosecond laser-assisted cataract surgery: a case report

**DOI:** 10.1186/s12886-017-0566-4

**Published:** 2017-09-16

**Authors:** Peiqing Chen, Yanan Zhu, Ke Yao

**Affiliations:** Eye Center of the 2nd Affiliated Hospital, School of Medicine, Zhejiang University, #88 Jiefang Road, Hangzhou, Zhejiang 310009 China

**Keywords:** Descemet Membrane Detachment, Femtosecond Laser, Cataract Surgery

## Abstract

**Background:**

Femtosecond laser-assisted cataract surgery (FLACS) has grown in popularity among ophthalmologists as a novel technique. However, descemet membrane detachment (DMD) began to be found as the complication after FLACS. We report a case of serious DMD following FLACS due to the inappropriate incision design.

**Case presentation:**

An 85-year-old man with apparent cornea arcus senilis underwent femtosecond laser-assisted cataract surgery in his right eye. A biplanar model was chosen for the main incision. A serious descemet membrane detachment (DMD) occurred at the end of phacoemulsification, which was connected with the main incision. However, the surgeon confused it with the transient swelling of corneal endothelium, and did not treated DMD timely. DMD was confirmed by anterior segment optical coherence tomography (AS-OCT) at the postoperative 1-month follow-up. Eventually DMD was resolved by intracameral perfluropropane (C3F8) gas injection.

**Conclusions:**

This case suggests that a careful incision separation and a triplanar incision design in FLACS may reduce the incidence of DMD in cataract surgery.

## Backgrounds

Femtosecond laser-assisted cataract surgery (FLACS) has become increasingly common since its introduction in 2008. Many benefits have been reported, including consistent and reproducible capsulotomy creation, watertight triplanar incisions, ability to correct astigmatism, less ultrasound energy, less endothelial cell loss, less macular edema, and better intraocular lens centration. However, some adverse effects have begun to be recognized, such as capsule tags and bridges, suction break, conjunctival hemorrhage, intraoperative miosis, and, less frequently, endothelial damage [1].

Descemet membrane detachment (DMD) is an infrequent complication following phacoemulsification. It can occur as a discontinuity or tear of the Descemet membrane, usually at or near the corneal incision. Reports established an incidence of 0.044% to 0.52% with the manual technique [2]. Recently, DMD was found to occur after FLACS [3]. Ricardo reported four cases of peri-incisional DMD due to air bubbles around the secondary incision. Here, we report on one case of serious DMD in FLACS that resulted from the incomplete incision and the inappropriate design of the main incision [4].

## Case presentation

**Fig. 1:**
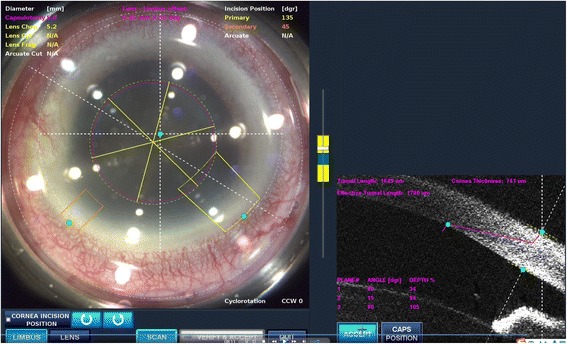
The main incision design of the patient in FLACS DMD occurred during surgery. Model: biplanar; laser energy:6 μJ; angulation:135°; focal spot separation: 5 μm; length: 1620 μm; width: 2 mm

**Fig. 2:**
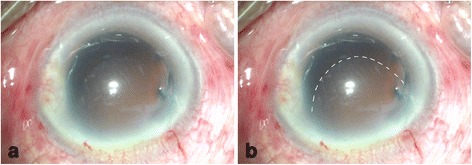
DMD happened during the surgery. **a** Status of DMD at the end of inspiration. **b** The dotted line shows the range of DMD

**Fig. 3:**
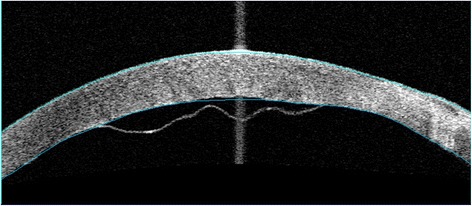
AS-OCT of planar DMD 1 month after phacoemulsification surgery

**Fig. 4:**
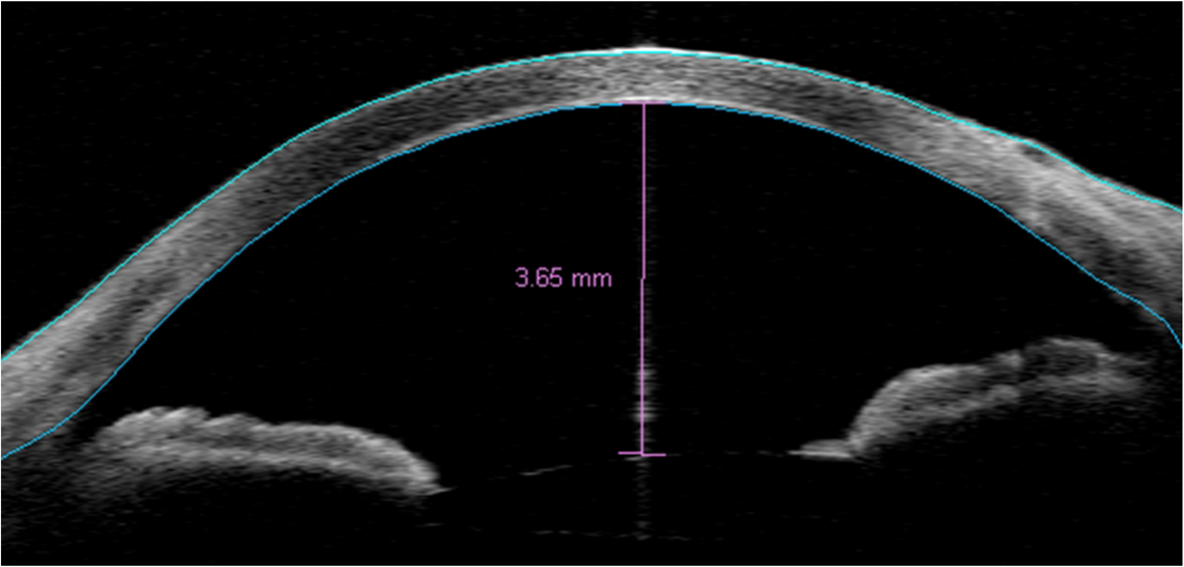
AS-OCT of resolved DMD after intracameral C3F8 gas injection

**Fig. 5:**
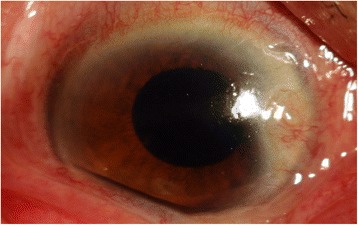
Anterior segment photograph of the patient 1 month after intracameral C3F8 gas injection

An 85-year-old man with apparent cornea arcus senilis underwent FLACS in the right eye. He had no systematic disease. The degree of nuclear hardness was IV, and his preoperative best-corrected visual acuity (BCVA) was 20/1000. In the process of femtosecond laser-assisted cataract surgery (LenSx Laser; Alcon Laboratories, Inc., Fort Worth, TX, USA), the surgeon chose a biplanar model for the main incision; the outer turning point was located at the 40% layer of cornea, while the inner turning point was located at the endothelium layer (Fig. 1). After the laser, the surgeon used a separator to separate the incision; however, it was somewhat difficult. Phacoemulsification was performed after separating the incision. A Stellaris phacoemulsificator (Bausch + Lomb Laboratories, Rochester, NY, USA) was used. The cumulative dissipated energy (CDE) was 35 s. DMD occurred at the end of phacoemulsification, which was located in the upper cornea and connected with the main incision. During aspiration, the range of DMD became larger (Fig. 2). Unfortunately, the surgeon confused it with the transient swelling of corneal endothelium, which could have resulted from the phacoemulsification energy. Therefore, the DMD was not treated during the surgery. The patient experienced severe corneal edema postoperatively. His BCVA was hand motion at the first day postoperatively, and 20/200 at the one-week follow-up. Considering that the corneal edema resulting from the phacoemulsification energy would last for a long time postoperatively, the surgeon did not perform anterior segment optical coherence tomography (AS-OCT) on the patient at the early postoperative stage (1 day and 1 week). A topical steroid (dexamethasone) and a nonsteroidal anti-inflammatory drug (NSAID) were prescribed for the patient, and each drug was given four times a day. However, after 1 month, limited corneal edema persisted in the central cornea, and her BCVA was 20/100. The AS-OCT (Carl Zeiss Meditec, Dublin, CA, USA) confirmed a typical DMD (Fig. 3). Therefore, we did an intracameral perfluropropane (C3F8) gas (14%) injection for her. In the surgery, 0.3 mL C3F8 gas was injected into the anterior chamer through the inferior temporal incision (5 o’ clock), and the aqueous humor was drained out through the inferior nasal incision (8 o’ clock) at the same time. Both of incisions were located at the transparent cornea area. Finally, the normal intraocular pressure was ensured at the close of surgery. The patient was informed and signed his consent according to the institutional guidelines and in compliance with the Helsinki Declaration. After gas injection for 1 month, the cornea was transparent, and the BCVA increased to 20/40 (Figs. 4, 5). The patient achieved a BCVA of 20/30 by the time the 6 month follow-up visit occurred.

## Discussion

DMD is an unusual complication of phacoemulsification surgery. In manual phacoemulsification surgery, the described surgical risk factors are three: first, incision-related, such as the use of dull blades [5], inappropriate incisions (oblique, excessively anterior, shelved incisions) [6], tight main incisions that do not fit the phaco probe [7]; second, instrument-related, such as the use of blunt instruments [8], inadvertent insertion of instruments between the corneal stroma and the Descemet membrane [9]; and, third, surgeon-related, such as engagement of the Descemet membrane during the irrigation/aspiration stage, unexpected injection of antibiotics, saline, or viscoelastic into the space between the deep stroma and the Descemet membrane [10], and surgeon inexperience [11]. After femtosecond laser was applied in cataract surgery, Ricardo first reported four localized DMD cases in FLACS that occurred because of an encapsulated bubble that did not spread into the anterior chamber (AC) and had formed when the incision was created [4]. Our eye center began to perform femtosecond laser-assisted cataract surgery in 2014. To date, we have performed more than 2000 of these surgeries. This was the first DMD case seen in our center.

Our case was a serious DMD after FLACS, and we analyzed the cause of the DMD. First, the patient had apparent cornea arcus senilis that may have influenced the laser penetration. In addition, the mild difficulty in separating the incision indicated the possibility of incomplete endothelium penetration. Also, the blunt force of the separation possibly made tiny dotted tears at the inner side of corneal incision. Furthermore, during phacoemulsification process, the weak endothelium became a support point for the phaco probe due to the biplanar incision design. Because of the frequent movement of the phaco probe and the irrigation, a serious DMD occurred at the end of the phacoemulsification. Moreover, a patient age of more than 65 years and the dense cataract of this case were also significant DMD risk factors, which have previously been seen [12].

Previous studies showed that inadequate docking, cornea arcus senilis, and corneal pannus may lead to incomplete laser corneal incisions in the femtosecond laser process [13, 14]. Here, our patient had apparent cornea arcus senilis, which rendered the incision imperfect. In the Chinese population, cornea arcus senilis is common. Thus, we suggest that the surgeons should be more aware when separating the incision in this type of patient.

**Fig. 6:**
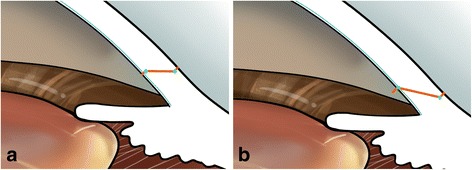
**a** Ideal triplanar incision design in FLACS. **b** Incision design in our case. Blue curve indicates Descemet membrane

**Fig. 7:**
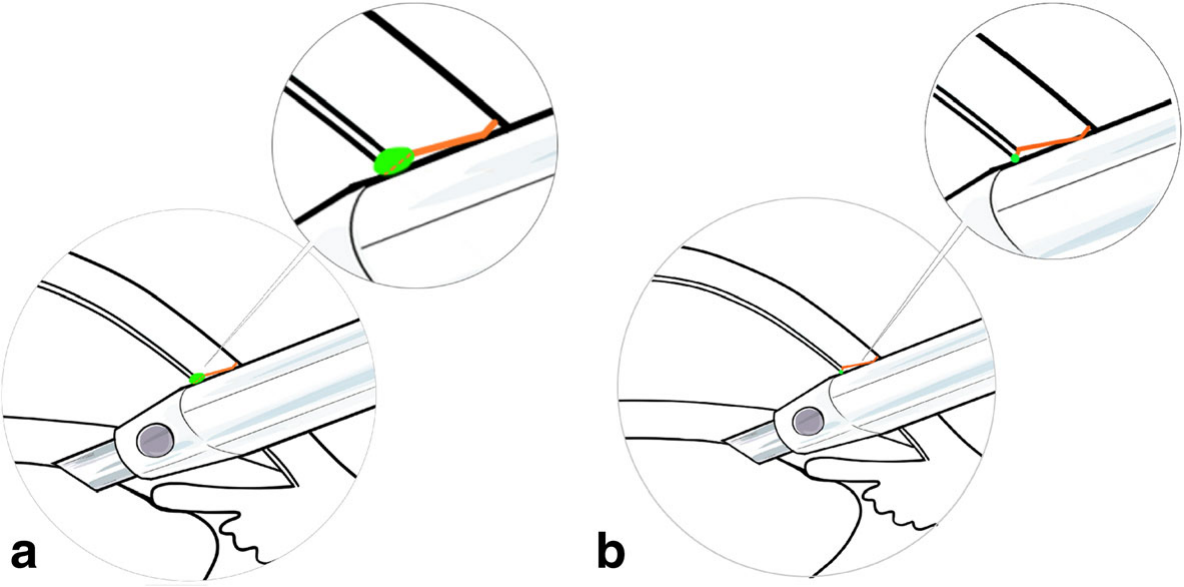
**a** The sketch shows that the endothelium and part of the stroma were affected by a well-distributed force of the instrument when a triplanar incision was made. **b** The sketch shows that the endothelium was affected by the force of the instrument only in our case. Green dots indicate the instrument’s support points

Additionally, we compared our biplanar incision with the ideal triplanar incision (Fig. 6). When a triplanar incision is performed, the endothelium and part of the stroma would be affected by a well-distributed force of instruments during the irrigation/aspiration stage (Fig. 7). However, in the current case, because of the biplanar incision design, only the endothelium was affected by the force, so the DMD readily occurred when the tiny tear existed. We therefore suggest that an ideal triplanar incision would reduce the incidence of DMD and make the incision watertight. The triplanar incision might be more suitable in FLACS; however, more clinical studies are required to confirm this.

DMDs usually arise from tears at the incision site that progress to the central cornea as aqueous humor enters the predescemetic space, and shallowness of the anterior chamber has been considered a predisposing factor [15]. It is particularly important that surgeons are aware of this. Sometimes, however, DMD is insidious. In our surgery, although the senior surgeon was highly experienced and had performed more than 10,000 surgeries, during the surgery, she mistook the DMD for transient corneal endothelial edema.

AS-OCT is a very useful tool in the diagnosis and classification of DMD and is becoming the standard method for diagnosis [16]. Unfortunately, considering that the corneal edema was due to the serious postoperative inflammation, we did not perform AS-OCT for the patient on the first postoperative day or at the one-week follow-up. However, at the 1-month follow-up, the patient was diagnosed with DMD by AS-OCT. Therefore, using AS-OCT at the early postsurgical stage could very possibly help the surgeon understand the status of the cornea, realize DMD as early as possible, and enable the patient to be treated in a timely manner. We suggest that in the presence of corneal edema in the postoperative setting, AS-OCT should be performed if it is available as slit-lamp examination of the posterior cornea and if DM may be unsatisfactory or incomplete due to opacification.

There is no gold standard for the treatment of DMDs. Many options have been described, such as medical treatment, manual reattachment with or without suturing, descemetopexy with air or expansible gases such as sulfur hexafluoride (SF6) or C3F8, and corneal transplantation, which could be penetrating keratoplasty, Descemet stripping endothelial keratoplasty, and, more recently, DMEK.2 In our case, considering that DMD existed for a long time (1 month), we used the intracameral C3F8 gas injection and were able to achieve a positive prognosis.

## Conclusion

Our case showed that the incomplete incision and inappropriate incision design became new risk factors for DMD in FLACS. It is important to carefully separate the incision and design a triplanar incision in FLACS to reduce the incidence of DMD in cataract surgery.

## Abbreviations

AC: Anterior chamber
AS-OCT: Anterior segment optical coherence tomography
BCVA: Best-corrected visual acuity
CDE: Cumulative dissipated energy
DMD: Descemet membrane detachment
FLACS: Femtosecond laser-assisted cataract surgery
NSAID: Nonsteroidal anti-inflammatory drug
